# Assessment of nausea across pregnancy and its association with maternal psychological status and perinatal outcomes: a prospective observational study in pregnant women

**DOI:** 10.1038/s41598-026-54721-8

**Published:** 2026-05-24

**Authors:** Tatsuya Yoshihara, Yasuhiko Okuda, Satoko Sasatsu, Eriko Ogasahara, Osamu Yoshino

**Affiliations:** Department of Obstetrics and Gynecology, University of Yamanashi, 1110 Shimokato, Chuo city, Yamanashi, 409-3898 Japan

**Keywords:** Maternal psychological status, Nausea, Perinatal outcomes, Pregnant women, Prospective observational study, Vomiting, Diseases, Health care, Medical research, Psychology, Psychology, Risk factors

## Abstract

Nausea during pregnancy is common, but previous studies have reported inconsistent findings regarding its associations with maternal mental health and perinatal complications. We assessed nausea across pregnancy using both subjective and quantitative measures and examined its associations with maternal mental health and perinatal outcomes. This prospective observational study included 424 pregnant women who received antenatal care and delivery management at our hospital between March 2024 and October 2025. Nausea was assessed at three gestational stages (early, mid, and late pregnancy) using (1) a three-level subjective assessment and (2) the Emesis Index (EI). We examined the associations of (i) nausea severity in early pregnancy, (ii) the presence of nausea at each gestational stage, and (iii) a nausea duration score (0–3) with maternal characteristics, mental health, and perinatal outcomes. Maternal mental health was evaluated using the State–Trait Anxiety Inventory (STAI) and the Patient Health Questionnaire-9 (PHQ-9). Group comparisons were performed using the Mann–Whitney U test, Kruskal–Wallis test, chi-square test, or Fisher’s exact test, as appropriate. In addition, multivariable linear regression analyses were performed to examine the associations between nausea and maternal psychological status after adjustment for maternal age, BMI, nulliparity, and history of psychiatric disorders. In both subjective and EI-based assessments, greater nausea severity in early pregnancy was associated with higher STAI-State and PHQ-9 scores. At each gestational stage, participants with nausea had significantly higher STAI-State and PHQ-9 scores than those without nausea. Higher nausea duration scores were also associated with a greater prevalence of psychiatric history and with higher STAI and PHQ-9 scores across pregnancy. In contrast, nausea showed little consistent association with most perinatal outcomes. The presence, greater severity, and persistence of nausea during pregnancy were associated with increased maternal anxiety and depressive symptoms. These findings suggest that persistent nausea may serve as a clinical indicator of increased psychological burden during pregnancy and support the need for greater attention to maternal mental health in women with ongoing symptoms.

**Supplementary Information:**

The online version contains supplementary material available at 10.1038/s41598-026-54721-8.

## Introduction

Nausea during pregnancy is an extremely common symptom, reported to be experienced by approximately 50–80% of pregnant women^1–3^. It typically manifests as nausea and vomiting of pregnancy between approximately 5 and 16 weeks of gestation and is generally considered to resolve spontaneously as pregnancy progresses^3,4^. Hyperemesis gravidarum, characterized by dehydration, electrolyte imbalance, and significant weight loss, can severely compromise the maternal general condition and may require hospitalization for treatment^4,5^. The prevalence of hyperemesis gravidarum has been reported to be approximately 0.5–2% of pregnant women^4–7^.

Previous studies have reported inconsistent findings regarding the association between nausea and vomiting of pregnancy and hyperemesis gravidarum and perinatal complications^8^. While some reports indicate that nausea and vomiting of pregnancy and hyperemesis gravidarum are not associated with preterm birth, fetal growth restriction (FGR), or hypertensive disorders of pregnancy (HDP)^9–11^, other studies have suggested that hyperemesis gravidarum may be associated with preterm birth, FGR, HDP, and even long-term neurodevelopmental outcomes in offspring^12–15^.

Most previous studies have focused on the presence or absence of nausea during early pregnancy and its association with perinatal complications^9,16^. In contrast, in routine clinical practice, a substantial proportion of pregnant women report that nausea persists beyond early pregnancy. Prolonged persistence of nausea may directly impair maternal quality of life, exacerbate psychological distress such as anxiety and depression, and potentially influence perinatal outcomes through maternal undernutrition. However, few studies have examined perinatal outcomes from a longitudinal perspective focusing on the duration of nausea.

In addition, several methods for assessing nausea have been proposed, including subjective evaluations based on patients’ perceived symptom burden and quantitative scoring systems based on the frequency of nausea and vomiting, such as the Emesis Index (EI) used in Japan and the Pregnancy-Unique Quantification of Emesis (PUQE) score^17,18^. Subjective assessment reflects the extent to which symptoms affect daily life, whereas quantitative tools provide a more standardized measure of symptom frequency and severity. Because these approaches may capture different aspects of the symptom experience, using both approaches may allow a more comprehensive evaluation of nausea across pregnancy. However, the degree of agreement between these approaches and their relative utility in predicting perinatal outcomes remain unclear.

Despite the high prevalence of nausea during pregnancy, an important knowledge gap remains regarding the clinical significance of nausea that persists beyond early pregnancy^1–3^. Previous studies have mainly focused on symptoms in early pregnancy and have often relied on a single assessment method^9,16^. Therefore, it remains unclear whether nausea that persists beyond early pregnancy has clinical relevance when evaluated across multiple gestational stages using both subjective and quantitative measures, particularly in relation to maternal psychological status and perinatal outcomes^8–10,16^. Clarifying these issues may improve understanding of the clinical significance of nausea beyond early pregnancy and may help identify women with persistent symptoms who could benefit from closer psychological assessment and supportive care during pregnancy.

In this study, we prospectively evaluated nausea using both subjective assessments and quantitative measures based on the EI at three time points—early, mid, and late pregnancy—among all pregnant women who underwent delivery management at our institution and examined its associations with maternal characteristics and perinatal outcomes. Specifically, this study aimed to examine three aspects: (1) the associations between the presence and severity of nausea in early pregnancy (5–16 weeks of gestation) and maternal characteristics and perinatal outcomes; (2) the characteristics of maternal background and perinatal outcomes according to the presence of nausea in mid (16–28 weeks of gestation) and late (≥ 29 weeks of gestation) pregnancy; and (3) the relationships between the duration of nausea and maternal characteristics and perinatal outcomes.

## Results

Among pregnant women who delivered at our institution during the study period, those who were evaluated at least once during early, mid, or late pregnancy were included in the analysis. A total of 424 participants were included in the final analysis.

1. To examine the associations between the severity of nausea in early pregnancy—assessed using subjective categories (A/B/C) and four EI-based severity groups—and maternal characteristics and perinatal outcomes. (Tables 1 and 2, supplementary Tables e1, 2)

**Table 1:** Maternal psychological status, and perinatal outcomes according to subjective nausea severity in early pregnancy. All participants who underwent subjective evaluation of nausea in early pregnancy (*n* = 224) are summarized. Nausea severity was classified into three groups based on subjective assessment: group A (not troubled by nausea), group B (symptoms present without interference with daily activities), and group C (symptoms interfering with daily activities). Data are presented as mean ± standard deviation or n (%). (*p < 0.05). GDM, gestational diabetes mellitus; PHQ-9, Patient Health Questionnaire-9; STAI, State-Trait Anxiety Inventory.

Gestational stage	0point (*n* = 58)	1point (*n* = 62)	2point (*n* = 8)	3point (*n* = 6)	*P* value
psychiatric disorders	6 (10%)	10 (16%)	4 (50%)	1 (17%)	0.04*
STAI-state in early pregnancy	43.6 ± 8.8	47.4 ± 10.0	52.5 ± 7.1	50.3 ± 14.0	0.04*
STAI-trait in early pregnancy	39.8 ± 8.3	42.6 ± 10.6	49.1 ± 11.7	46.0 ± 12.7	0.09
PHQ-9 in early pregnancy	5.5 ± 3.9	8.1 ± 4.8	15.2 ± 3.5	11.4 ± 7.3	< 0.001*
STAI-state in mid pregnancy	38.8 ± 8.7	40.5 ± 10.6	48.8 ± 5.6	44.7 ± 8.4	0.009*
STAI-trait in mid pregnancy	36.8 ± 9.2	38.5 ± 11.6	49.0 ± 7.6	42.5 ± 10.3	0.02*
PHQ-9 in mid pregnancy	3.6 ± 2.9	5.4 ± 4.0	8.3 ± 4.2	8.5 ± 5.9	0.003*
STAI-state in late pregnancy	38.1 ± 9.3	39.3 ± 9.2	49.6 ± 7.3	40.0 ± 13.5	0.02*
STAI-trait in late pregnancy	36.6 ± 9.7	37.5 ± 9.9	46.8 ± 8.6	38.7 ± 12.3	0.04*
PHQ-9 in late pregnancy	4.2 ± 3.1	5.4 ± 3.4	7.4 ± 4.0	6.3 ± 6.4	0.06

**Table 2:** Maternal psychological status, and perinatal outcomes according to EI-based nausea severity in early pregnancy. All participants who underwent quantitative evaluation using the Emesis Index (EI) in early pregnancy (*n* = 212) are summarized. Nausea severity was categorized into four groups based on EI scores: no symptoms (0–3), mild (4–5), moderate (6–10), and severe (≥ 11). Data are presented as mean ± standard deviation or n (%). (*p < 0.05) GDM, gestational diabetes mellitus; PHQ-9, Patient Health Questionnaire-9; STAI, State-Trait Anxiety Inventory.

	A (*n* = 30)	B (*n* = 129)	C (*n* = 65)	*P* value
STAI-state in early pregnancy	41.2 ± 10.4	45.4 ± 9.0	49.6 ± 10.2	0.002*
STAI-trait in early pregnancy	37.8 ± 8.9	42.2 ± 9.8	43.8 ± 11.3	0.03*
PHQ-9 in early pregnancy	3.7 ± 4.9	6.5 ± 4.0	11.0 ± 4.9	< 0.001*
GDM	6 (20%)	10 (8.1%)	3 (4.7%)	0.04*

Among the 224 participants who underwent subjective evaluation of nausea in early pregnancy, the distribution of nausea severity was as follows: group A (not troubled by nausea symptoms), 30 cases (13%); group B (symptoms present without interference with daily activities), 129 cases (58%); and group C (symptoms interfering with daily activities), 65 cases (29%). Quantitative evaluation using the EI was performed in 212 participants, of whom 101 (47%) were classified as having no symptoms (0–3 points), 67 (31%) as mild (4–5 points), 47 (21%) as moderate (6–10 points), and 2 (1%) as severe (≥ 11 points).

With respect to maternal characteristics, no significant differences were observed among groups in either the subjective or quantitative assessments. Regarding psychological status, participants in group C, who experienced the most severe symptoms, had significantly higher scores than the other groups for state anxiety on the State–Trait Anxiety Inventory (STAI) (A: 41.2 ± 10.4 vs. B: 45.4 ± 9.0 vs. C: 49.6 ± 10.2; *p* = 0.002), trait anxiety on the STAI (37.8 ± 8.9 vs. 42.2 ± 9.8 vs. 43.8 ± 11.3; *p* = 0.03), and PHQ-9 (3.7 ± 4.9 vs. 6.5 ± 4.0 vs. 11.0 ± 4.9; *p* < 0.001). Similarly, in the quantitative assessment, increasing severity of nausea was associated with progressively higher STAI state anxiety scores (no symptoms: 43.5 ± 9.2 vs. mild: 46.5 ± 8.8 vs. moderate: 49.6 ± 11.5 vs. severe: 50.0 ± 7.1; *p* = 0.01) and PHQ-9 scores (5.2 ± 3.9 vs. 7.7 ± 4.0 vs. 11.4 ± 5.5 vs.18.0 ± 0; *p* < 0.001).

With respect to perinatal outcomes, the frequency of gestational diabetes mellitus (GDM) was significantly higher in group A in the subjective assessment (20% vs. 8.1% vs. 4.7%; *p* = 0.04). In the quantitative assessment, a significant between-group difference was observed in the rate of neonatal intensive care unit (NICU) admission (19% vs. 5.0% vs. 24% vs. 0%; *p* = 0.03). No significant differences were observed in other perinatal complications or neonatal outcomes according to the severity of nausea.

In multivariable linear regression analyses adjusted for maternal age, BMI, nulliparity, and history of psychiatric disorders, nausea severity remained significantly associated with higher STAI-State scores (Regression coefficient = 4.295, 95% confidence intervals (CI) 2.355–6.235, *p* < 0.001) and Patient Health Questionnaire-9 (PHQ-9) scores (Regression coefficient = 3.622, 95% CI 2.383–4.862, *p* < 0.001) (Supplementary Table 3).

(2) To categorize nausea status at each gestational stage (early, mid, and late pregnancy) into “no symptoms” (subjective evaluation: A; EI ≤ 3) and “symptoms present” (subjective evaluation: B + C; EI ≥ 4), and to evaluate maternal characteristics and perinatal outcomes separately using subjective assessment and EI. (Tables 3 and 4, Supplementary Tables 4–9).

**Table 3:** Anxiety and depressive symptoms according to the presence of nausea at each gestational stage based on subjective assessment.

	No symptoms (*n* = 101)	Mild (*n* = 67)	Moderate (*n* = 47)	Severe (*n* = 2)	*P* value
STAI-state in early pregnancy	43.5 ± 9.2	46.5 ± 8.8	49.6 ± 11.5	50.0 ± 7.1	0.01*
STAI-trait in early pregnancy	40.4 ± 8.7	43.1 ± 10.0	43.1 ± 12.5	41.5 ± 10.6	0.31
PHQ-9 in early pregnancy	5.2 ± 3.9	7.7 ± 4.0	11.4 ± 5.5	18.0 ± 0	< 0.001*
GDM	8 (8.2%)	8 (13%)	2 (4.4%)	0	0.40

Participants were categorized according to the presence of nausea at each gestational stage based on subjective assessment: “no symptoms” (category A) and “symptoms present” (categories B + C). Anxiety and depressive symptoms were evaluated using the State–Trait Anxiety Inventory (STAI) and the Patient Health Questionnaire-9 (PHQ-9). Data are presented as mean ± standard deviation. (*p < 0.05)

PHQ-9, Patient Health Questionnaire-9; STAI, State–Trait Anxiety Inventory.

**Table 4:** Anxiety and depressive symptoms according to the presence of nausea at each gestational stage based on the Emesis Index.

Gestational stage		No symptoms	Symptoms present	*P* value
Early pregnancy		*n* = 30	*n* = 194	
	STAI-state	41.2 ± 10.4	46.8 ± 9.6	0.02*
	STAI-trait	37.8 ± 8.9	42.8 ± 10.3	0.02*
	PHQ-9	3.7 ± 4.9	7.9 ± 4.7	< 0.001*
Mid pregnancy		*n* = 307	*n* = 95	
	STAI-state	40.0 ± 8.9	44.4 ± 9.3	< 0.001*
	STAI-trait	37.4 ± 9.5	42.1 ± 10.4	< 0.001*
	PHQ-9	4.3 ± 3.5	6.5 ± 4.9	< 0.001*
Late pregnancy		*n* = 324	*n* = 100	
	STAI-state	39.6 ± 8.9	41.8 ± 9.4	0.03*
	STAI-trait	36.8 ± 9.8	39.3 ± 9.7	0.02*
	PHQ-9	4.4 ± 3.5	6.1 ± 3.9	0.003*

Participants were categorized based on EI scores at each gestational stage into “no symptoms” (EI ≤ 3) and “symptoms present” (EI ≥ 4). Anxiety and depressive symptoms were assessed using STAI and PHQ-9. Data are presented as mean ± standard deviation. (*p < 0.05)

EI, Emesis Index; PHQ-9, Patient Health Questionnaire-9; STAI, State–Trait Anxiety Inventory.

Based on subjective assessment, nausea was reported in 87% of participants during early pregnancy, 24% during mid-pregnancy, and 24% during late pregnancy. In contrast, quantitative assessment identified symptoms in 52.4%, 8.3%, and 6.2% of participants during early, mid-, and late pregnancy, respectively.

With respect to maternal characteristics, no significant differences in physical background factors were observed according to the presence or absence of nausea at any gestational stage, including early, mid, or late pregnancy. In contrast, regarding psychological status, subjective assessment revealed that during early pregnancy, participants with nausea had significantly higher STAI state anxiety scores (no symptoms: 41.2 ± 10.4 vs. symptoms present: 46.8 ± 9.6; *p* = 0.02), STAI trait anxiety scores (37.8 ± 8.9 vs. 42.8 ± 10.3; *p* = 0.02), and PHQ-9 scores (3.7 ± 4.9 vs. 7.9 ± 4.7; *p* < 0.001) compared with those without symptoms. This pattern was consistent across both nausea assessment methods, with higher STAI and PHQ-9 scores observed among participants with nausea during both mid and late pregnancy.

Regarding perinatal and neonatal outcomes, GDM was significantly less frequent among participants with nausea based on subjective assessment in early pregnancy (no symptoms: 20% vs. symptoms present: 6.9%; *p* = 0.02). In addition, postpartum hemorrhage (PPH) was significantly more frequent among participants with nausea based on quantitative assessment in mid-pregnancy (43% vs. 64%; *p* = 0.03). No significant differences were observed in other perinatal outcomes.

Multivariable linear regression analyses confirmed significant associations between nausea presence and higher STAI-State and PHQ-9 scores in early pregnancy (STAI-State: Regression coefficient = 5.557, 95% CI 1.912–9.202, *p* = 0.003; PHQ-9: Regression coefficient = 4.200, 95% CI 1.808–6.593, *p* < 0.001) and mid-pregnancy (STAI-State: Regression coefficient = 4.187, 95% CI: 2.101–6.273, *p* < 0.001; PHQ-9: Regression coefficient = 1.879, 95% CI 0.713–3.046, *p* = 0.002), after adjustment for maternal age, BMI, nulliparity, and psychiatric history. In late pregnancy, nausea remained significantly associated with higher PHQ-9 scores (Regression coefficient = 1.276, 95% CI 0.282–2.271, *p* = 0.012), whereas the association with STAI-State scores did not reach statistical significance (*p* = 0.060). (Supplementary Table 10).

(3) To score the duration of nausea based on its presence across early, mid, and late pregnancy using both subjective evaluation and EI, and to examine the associations between the duration of nausea within the same individual and maternal characteristics and perinatal outcomes. (Tables 5 and 6, Supplementary Tables 11,12)

**Table 5:** Anxiety and depressive symptoms according to the duration score of nausea based on subjective assessment.

Gestational stage		No symptoms	Symptoms present	*P* value
Early pregnancy		*n* = 101	*n* = 111	
	STAI-state	43.5 ± 9.2	47.8 ± 10.1	0.002*
	STAI-trait	40.4 ± 8.7	43.1 ± 11.0	0.06
	PHQ-9	5.2 ± 3.9	9.3 ± 5.0	< 0.001*
Mid pregnancy		*n* = 330	*n* = 30	
	STAI-state	40.8 ± 9.5	45.0 ± 8.8	0.01*
	STAI-trait	38.3 ± 10.1	43.8 ± 10.5	0.01*
	PHQ-9	4.5 ± 3.7	8.4 ± 5.3	0.01*
Late pregnancy		*n* = 362	*n* = 24	
	STAI-state	39.7 ± 9.0	44.5 ± 11.1	0.03*
	STAI-trait	37.1 ± 9.8	42.5 ± 12.1	0.02*
	PHQ-9	4.6 ± 3.4	7.6 ± 4.9	0.01*

This analysis included participants who underwent continuous assessment of nausea throughout pregnancy (*n* = 134). The duration score (range, 0–3) was calculated by assigning one point for the presence of nausea at each gestational stage (early, mid, and late pregnancy), defined as subjective assessment categories B or C. Anxiety and depressive symptoms were assessed using the State–Trait Anxiety Inventory (STAI) and the Patient Health Questionnaire-9 (PHQ-9). Data are presented as mean ± standard deviation or n (%). (*p < 0.05)

PHQ-9, Patient Health Questionnaire-9; STAI, State–Trait Anxiety Inventory.

**Table 6:** Anxiety and depressive symptoms according to the duration score of nausea based on the Emesis Index.

Gestational stage	0point (*n* = 14)	1point (*n* = 74)	2point (*n* = 28)	3point (*n* = 18)	*P* value
Psychiatric disorders	1 (7.1%)	8 (11%)	5 (18%)	7 (39%)	0.02
STAI-state in early pregnancy	41.1 ± 11.4	45.2 ± 9.1	48.4 ± 9.0	50.6 ± 10.4	0.05
STAI-trait in early pregnancy	37.6 ± 8.7	40.6 ± 9.7	44.5 ± 9.2	46.7 ± 11.2	0.03*
PHQ-9 in early pregnancy	4.0 ± 5.4	6.9 ± 4.1	9.1 ± 5.6	11.8 ± 5.8	0.003*
STAI-state in mid pregnancy	37.6 ± 9.8	38.2 ± 9.0	43.2 ± 8.2	47.4 ± 10.9	0.001*
STAI-trait in mid pregnancy	34.6 ± 10.2	36.1 ± 9.9	42.5 ± 9.1	45.9 ± 11.7	0.001*
PHQ-9 in mid pregnancy	2.9 ± 3.5	4.2 ± 3.1	5.5 ± 3.9	8.8 ± 4.8	< 0.001*
STAI-state in late pregnancy	38.4 ± 11.4	37.7 ± 8.4	42.3 ± 9.7	42.7 ± 11.5	0.08
STAI-trait in late pregnancy	35.8 ± 11.9	36.1 ± 9.3	40.3 ± 9.5	41.6 ± 11.1	0.60
PHQ-9 in late pregnancy	3.3 ± 3.2	4.6 ± 2.5	6.1 ± 4.4	6.4 ± 4.8	0.09

This analysis included participants who underwent continuous quantitative assessment using the Emesis Index (EI) throughout pregnancy (*n* = 134). The duration score (range, 0–3) was calculated by assigning one point for the presence of nausea at each gestational stage, defined as an EI score ≥ 4. Anxiety and depressive symptoms were evaluated using STAI and PHQ-9. Data are presented as mean ± standard deviation or n (%). (*p < 0.05)

EI, Emesis Index; PHQ-9, Patient Health Questionnaire-9; STAI, State–Trait Anxiety Inventory.

A total of 134 participants who underwent continuous assessment of nausea throughout the entire pregnancy were included in this analysis. To examine the impact of the duration of nausea on perinatal outcomes, a duration score (range 0–3) was created by assigning one point for the presence of nausea at each gestational stage—early, mid, and late pregnancy—defined as subjective evaluation categories B or C and/or an EI score ≥ 4. Based on subjective assessment, the distribution of the duration score was as follows: 14 participants (10%) with a score of 0 (no symptoms throughout pregnancy), 74 (55%) with a score of 1 (symptoms in one gestational stage only), 28 (21%) with a score of 2 (symptoms in two stages), and 18 (13%) with a score of 3 (symptoms throughout pregnancy). In the quantitative assessment, 58 participants (43%) had a score of 0, 62 (46%) had a score of 1, 8 (6.0%) had a score of 2, and 6 (4.5%) had a score of 3.

**Fig. 1:**
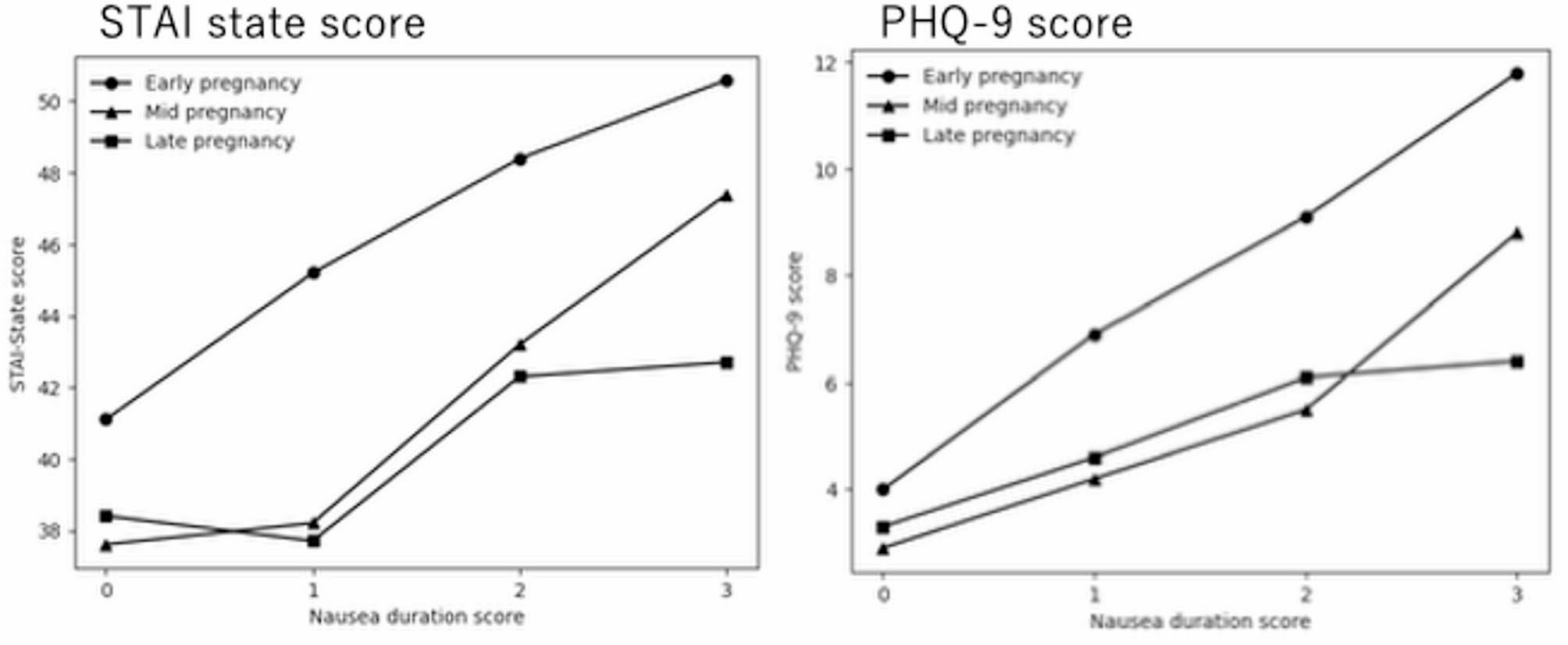
Association between duration of nausea and psychological scores based on subjective assessment. This analysis included participants who underwent continuous assessment of nausea throughout pregnancy (*n* = 134). The nausea duration score (range, 0–3) was calculated by assigning one point for the presence of nausea at each gestational stage (early, mid, and late pregnancy), defined as subjective assessment categories B or C. STAI state and PHQ-9 scores according to the nausea duration score. Data are presented as mean values. Statistical analyses were performed using the Kruskal–Wallis test. PHQ-9, Patient Health Questionnaire-9; STAI, State–Trait Anxiety Inventory.

In the subjective assessment, a higher nausea duration score was significantly associated with a greater prevalence of a history of psychiatric disorders (score 0: 7.1% vs. score 1: 11% vs. score 2: 18% vs. score 3: 39%; *p* = 0.02). Furthermore, analysis of psychological status demonstrated that increasing nausea duration scores were associated with significantly higher STAI state, trait and PHQ-9 score. STAI state and PHQ-9 scores were significantly associated with nausea duration across early, mid, and late pregnancy (Fig. 1)

Similarly, in the quantitative assessment, a significant association was observed between the nausea duration score and a history of psychiatric disorders (10% vs. 16% vs. 50% vs. 17%; *p* = 0.04). Further analysis of psychological status demonstrated significant associations between the nausea duration score and STAI state and PHQ-9 scores across early, mid, and late pregnancy.

No significant associations were observed between the nausea duration score and perinatal or neonatal outcomes in either the subjective or quantitative assessments.

In multivariable linear regression analyses, nausea duration score was significantly associated with higher STAI-State scores in early pregnancy (Regression coefficient = 2.403, 95% CI 0.447–4.359, *p* = 0.016) and mid-pregnancy (Regression coefficient = 3.275, 95% CI 1.377–5.174, *p* < 0.001), but not in late pregnancy. Nausea duration score was also significantly associated with higher PHQ-9 scores at all three gestational stages (early: Regression coefficient = 2.018, *p* = 0.003; mid: Regression coefficient = 1.603, *p* < 0.001; late: Regression coefficient = 0.800, *p* = 0.026), after adjustment for maternal age, BMI, nulliparity, and psychiatric history (Supplementary Table 13).

## Discussion

In this study, we assessed nausea at three gestational stages and examined its relationships with perinatal outcomes and maternal psychological status. As a key finding, the presence, severity, and duration of nausea showed little direct association with the occurrence of major perinatal complications, whereas they remained significantly associated with maternal psychological burden across multiple gestational stages, even after adjustment for potential confounders including psychiatric history.

In particular, among participants in whom nausea persisted throughout pregnancy based on subjective assessment, the prevalence of a history of psychiatric disorders approached 40%, and STAI and PHQ-9 scores remained consistently elevated from early to late pregnancy. This finding suggests a potential vicious cycle in which prolonged physical discomfort reduces quality of life and increases psychological burden, thereby exacerbating depressive symptoms. Furthermore, a bidirectional relationship may be postulated, whereby pregnant women with pre-existing psychological vulnerability are more likely to experience more severe and prolonged nausea as a somatic manifestation^19,20^. In other words, the worsening and prolonged persistence of nausea may function not merely as an extension of a physiological phenomenon, but as a “somatic signal” that brings underlying mental health issues to clinical attention^21^.

On the other hand, even when nausea became severe or prolonged, it did not have a substantial adverse impact on perinatal outcomes. From a clinical perspective, this finding may provide reassurance to pregnant women suffering from nausea, indicating that excessive concern regarding perinatal outcomes may be unnecessary.

However, it should also be noted that the favorable perinatal outcomes observed in this study may paradoxically contribute to increased psychological isolation among pregnant women. The finding that nausea is not associated with adverse perinatal outcomes may lead healthcare providers and others to regard it as a symptom that does not warrant significant attention. For the individuals experiencing nausea, the difficulty in translating their suffering into objective measures or clinical complications may lead to feelings of being underestimated or insufficiently understood by others^2,22^. This discrepancy between subjective experience and objective assessment may partly underlie the elevated levels of anxiety and depression reflected in the STAI and PHQ-9 scores^23^.

With respect to individual perinatal complications, we found that the incidence of GDM was significantly lower among participants who experienced nausea in early pregnancy. This finding may be attributable to a protective effect of reduced energy intake associated with nausea, as well as to the potential influence of the hormonal milieu in early pregnancy on glucose metabolism^24,25^. In addition, an association between nausea and PPH was suggested based on quantitative assessment in mid-pregnancy. Previous studies have also suggested associations between hyperemesis gravidarum and abnormalities in the coagulation system or vascular fragility, and the involvement of nutritional imbalance cannot be excluded^10^. However, further investigation with larger sample sizes is required to clarify these relationships.

Several limitations of this study should be acknowledged. First, this was a single-center study with a relatively limited sample size. Second, although this was a prospective study based on questionnaire assessments, detailed dietary intake data and longitudinal data on patterns of gestational weight change were not systematically collected as part of the study protocol. Therefore, we were unable to evaluate the relationships among nausea severity, nutritional intake, and weight change over time.

By evaluating nausea across pregnancy, this study highlights the potential clinical importance of persistent nausea as an indicator of increased psychological burden during pregnancy. Obstetricians should not regard nausea and vomiting of pregnancy as merely a transient condition that resolves spontaneously; rather, particularly in women with severe or persistent symptoms, attention should also be paid to underlying anxiety and depressive symptoms, and appropriate psychological assessment and supportive care may be warranted. Further studies with larger sample sizes and more detailed assessment of nutritional intake and weight change are needed to clarify the broader clinical significance of persistent nausea and its relationship with perinatal outcomes.

## Methods

### Study design and participants

This prospective observational study included pregnant women who received antenatal care and delivery management at our institution between March 2024 and October 2025. This manuscript was prepared in accordance with the STROBE guidelines for observational studies. Eligible participants were all women who underwent nausea assessment at least once during early pregnancy (5–16 weeks of gestation), mid-pregnancy (16–28 weeks of gestation), or late pregnancy (≥ 29 weeks of gestation). Multiple pregnancies were excluded from the analysis. Because this study was based on the consecutively available population at our institution during the study period, no formal a priori sample size calculation was performed.

### Nausea assessment

During pregnancy, interviews regarding nausea were conducted at three time points: early pregnancy, mid-pregnancy, and late pregnancy. Nausea was assessed using two approaches: a subjective evaluation and a quantitative evaluation. The subjective evaluation was intended to capture the participant’s perceived burden of symptoms in daily life, whereas the quantitative evaluation using the EI was used to provide a more standardized assessment of symptom frequency and severity. For the subjective evaluation, participants were asked to self-report their symptoms by selecting one of three categories: (A) not troubled by nausea symptoms; (B) experiencing symptoms without interference with daily activities; or (C) experiencing symptoms that interfere with daily activities. For the quantitative evaluation, the EI was calculated based on questionnaire items assessing the frequency of nausea and vomiting as well as loss of appetite, salivation, and oral discomfort (Supplementary Table 14). Each EI item was scored from 0 to 3, with higher total scores indicating more severe symptoms. EI scores were categorized as follows: 0–3 points as no symptoms, 4–5 points as mild, 6–10 points as moderate, and ≥ 11 points as severe.

### Study outcomes and data collection

The primary analyses of this study comprised the following three components.

1. To examine the associations between the severity of nausea in early pregnancy—assessed using subjective categories (A/B/C) and four EI-based severity groups—and maternal characteristics and perinatal outcomes.
2. To categorize nausea status at each gestational stage (early, mid, and late pregnancy) into “no symptoms” (subjective evaluation: A; EI ≤ 3) and “symptoms present” (subjective evaluation: B + C; EI ≥ 4), and to evaluate maternal characteristics and perinatal outcomes separately using subjective assessment and EI.
3. To score the duration of nausea based on its presence across early, mid, and late pregnancy using both subjective evaluation and EI, and to examine the associations between the duration of nausea within the same individual and maternal characteristics and perinatal outcomes.

Maternal characteristics collected from the medical records included maternal age, pre-pregnancy body mass index, gestational weight gain, parity, use of assisted reproductive technology, and a history of psychiatric disorders. Maternal psychological status was assessed using self-administered questionnaires for anxiety and depression, namely the STAI and the PHQ-9. Perinatal data collected from the medical records included gestational age at delivery, preterm birth, HDP, GDM, FGR, cesarean delivery, emergency cesarean delivery, intrapartum blood loss, PPH, and placental weight. Neonatal outcomes assessed included birth weight, Apgar scores at 1 and 5 min, umbilical artery blood pH, and NICU admission.

STAI is a self-administered questionnaire consisting of 20 items each that assesses anxiety from two dimensions: state anxiety and trait anxiety. Scores for state anxiety and trait anxiety each range from 20 to 80, with higher scores indicating greater levels of anxiety. In previous studies, STAI-State scores around 40 have been used as an approximate indicator of elevated anxiety^26,27^. In the present study, STAI scores were analyzed as continuous variables. PHQ-9 is a self-administered scale consisting of nine items that assesses the frequency of depressive symptoms experienced over the preceding two weeks, with total scores ranging from 0 to 27, with higher scores indicating more severe depressive symptoms. A previous meta-analysis reported that PHQ-9 cut-off values around 8–11 have acceptable diagnostic properties for identifying depression^28^. PHQ-9 scores were also analyzed as continuous variables in this study. Both instruments are widely used and established tools for the assessment of anxiety and depressive symptoms. FGR was defined as an estimated fetal weight below − 1.5 standard deviations. Emergency cesarean delivery was defined as cesarean delivery performed for non-reassuring fetal status. PPH was defined as blood loss of ≥ 500 g after vaginal delivery or ≥ 1000 g after cesarean delivery.

### Ethical considerations

This study was approved by the institutional ethics committee of the University of Yamanashi (Approval No. 2821). The study was conducted in accordance with relevant ethical guidelines and regulations. Information regarding the study was disclosed on the hospital website, and informed consent was obtained using an opt-out approach. Clinical data were collected from medical records under institutional ethical approval, and patient confidentiality was protected throughout the study.

### Statistical analysis

Continuous variables were expressed as mean ± standard deviation, and between-group comparisons were performed using the Mann–Whitney U test and the Kruskal–Wallis test. Categorical variables were presented as frequencies (percentages) and compared using the chi-square test or Fisher’s exact test, as appropriate. A p-value of < 0.05 was considered statistically significant. To further examine the associations between nausea and maternal psychological status, multivariable linear regression analyses were performed with STAI-State and PHQ-9 scores as dependent variables. Covariates were selected a priori based on clinical relevance and included maternal age, BMI, nulliparity, and history of psychiatric disorders. Statistical analyses were performed using JMP Pro version 18.2.0.

## Supplementary Information

Below is the link to the electronic supplementary material.

Supplementary Material 1

## Data Availability

The data that support the findings of this study are available on request from the corresponding author. The data are not publicly available due to their containing information that could compromise the privacy of research participants.
