# Supplementary material for: Assessment of nausea across pregnancy and its association with maternal psychological status and perinatal outcomes: a prospective observational study in pregnant women
Source: Sci Rep. 2026 May 24;16:23690. doi: 10.1038/s41598-026-54721-8 (PMC13424093; doi:10.1038/s41598-026-54721-8)
Supplement: Supplementary file 1 — Supplementary Material 1 [file 41598_2026_54721_MOESM1_ESM.docx]

**Supplementary Table 1:** Maternal characteristics, psychological status, and perinatal outcomes according to subjective nausea severity in early pregnancy.

All participants who underwent subjective evaluation of nausea in early pregnancy (n = 224) are summarized. Nausea severity was classified into three groups based on subjective assessment: group A (not troubled by nausea), group B (symptoms present without interference with daily activities), and group C (symptoms interfering with daily activities). Data are presented as mean ± standard deviation or n (%). (*p<0.05)

ART, assisted reproductive technology; BMI, body mass index; FGR, fetal growth restriction; HDP, hypertensive disorders of pregnancy; NICU, neonatal intensive care unit; PHQ-9, Patient Health Questionnaire-9; PPH, postpartum hemorrhage; STAI, State-Trait Anxiety Inventory

|  | A (n=30) | B (n=129) | C (n=65) | P value |
| --- | --- | --- | --- | --- |
| Age | 33.6±4.5 | 34.7±5.0 | 33.4±5.1 | 0.25 |
| Pre-pregnancy BMI (kg/m^2^) | 21.3±3.0 | 21.9±3.8 | 23.2±5.3 | 0.43 |
| Gestational weight gain (kg) | 6.7±4.1 | 10.4±4.2 | 10.9±4.4 | 0.27 |
| primipara | 17 (57%) | 68 (51%) | 34 (52%) | 0.86 |
| ART | 9 (30%) | 43 (33%) | 20 (30%) | 0.90 |
| Psychiatric disorders | 4 (13%) | 21 (16%) | 9 (14%) | 0.86 |
| Gestational age at delivery (week) | 38.3±1.1 | 38.3±1.4 | 38.7±1.2 | 0.11 |
| Preterm birth | 0 | 6 (4.8%) | 3 (4.7%) | 0.47 |
| HDP | 7 (23%) | 17 (14%) | 8 (13%) | 0.47 |
| FGR | 2 (6.7%) | 10 (8.1%) | 1 (1.6%) | 0.20 |
| Cesarean section | 12 (40%) | 46 (37%) | 23 (36%) | 0.93 |
| Emergency cesarean section | 2 (6.7%) | 7 (5.7%) | 2 (3.1%) | 0.68 |
| Intrapartum blood loss (mL) | 644±402 | 706±407 | 779±487 | 0.27 |
| PPH | 9 (30%) | 52 (42%) | 31 (48%) | 0.24 |
| Placental weight (g) | 511±88 | 560±93 | 563±107 | 0.63 |
| Birth weight (g) | 2988±401 | 2939±382 | 3050±345 | 0.16 |
| Apgar score at 1 minute | 8.2±0.8 | 8.1±1.3 | 8.1±1.0 | 0.33 |
| Apgar score at 5 minutes | 9.3±0.5 | 9.3±0.6 | 9.3±0.4 | 0.39 |
| Umbilical artery blood pH | 7.30±0.07 | 7.29±0.06 | 7.30±0.06 | 0.69 |
| NICU admission | 4 (13%) | 23 (18%) | 7 (11%) | 0.38 |

**Supplementary Table 2. Maternal characteristics, psychological status, and perinatal outcomes according to EI-based nausea severity in early pregnancy**
All participants who underwent quantitative evaluation using the Emesis Index (EI) in early pregnancy (n = 212) are summarized. Nausea severity was categorized into four groups based on EI scores: no symptoms (0–3), mild (4–5), moderate (6–10), and severe (≥11). Data are presented as mean ± standard deviation or n (%). (*p<0.05)

ART, assisted reproductive technology; BMI, body mass index; FGR, fetal growth restriction; HDP, hypertensive disorders of pregnancy; NICU, neonatal intensive care unit; PHQ-9, Patient Health Questionnaire-9; PPH, postpartum hemorrhage; STAI, State-Trait Anxiety Inventory

|  | No symptoms (n=101) | Mild (n=67) | Moderate (n=47) | Severe (n=2) | P value |
| --- | --- | --- | --- | --- | --- |
| Age | 35.0±4.9 | 34.4±4.6 | 32.6±5.3 | 31.0±1.4 | 0.08 |
| Pre-pregnancy BMI (kg/m^2^) | 21.7±3.5 | 22.1±3.6 | 23.5±6.1 | 28.4±4.6 | 0.23 |
| Gestational weight gain (kg) | 11.1±3.8 | 9.9±4.2 | 10.8±4.8 | 4.8±3.4 | 0.19 |
| primipara | 56 (55%) | 28 (45%) | 23 (49%) | 2 (100%) | 0.30 |
| ART | 35 (35%) | 23 (37%) | 13 (28%) | 1 (50%) | 0.72 |
| Psychiatric disorders | 16 (16%) | 6 (9.7%) | 10 (21%) | 0 | 0.36 |
| Gestational age at delivery (week) | 38.4±1.4 | 38.5±1.0 | 38.4±1.6 | 39.0±0 | 0.90 |
| Preterm birth | 5 (5.1%) | 0 | 4 (8.7%) | 0 | 0.17 |
| HDP | 14 (14%) | 8 (13%) | 9 (20%) | 0 | 0.74 |
| FGR | 5 (5.1%) | 4 (6.7%) | 3 (6.5%) | 0 | 0.95 |
| Cesarean section | 40 (41%) | 19 (32%) | 16 (35%) | 0 | 0.46 |
| Emergency cesarean section | 5 (5.1%) | 2 (3.4%) | 2 (4.4%) | 0 | 0.95 |
| Intrapartum blood loss (mL) | 670±362 | 733±458 | 763±504 | 504±120 | 0.79 |
| PPH | 36 (37%) | 29 (48%) | 21 (46%) | 1 (50%) | 0.49 |
| Placental weight (g) | 541±87 | 558±91 | 591±114 | 463±12 | 0.09 |
| Birth weight (g) | 2940±379 | 3021±337 | 2997±420 | 2815±95 | 0.44 |
| Apgar score at 1 minute | 8.1±1.2 | 8.3±0.8 | 8.0±1.3 | 8.5±0.7 | 0.78 |
| Apgar score at 5 minutes | 9.3±0.6 | 9.3±0.5 | 9.3±0.6 | 10±0 | 0.25 |
| Umbilical artery blood pH | 7.29±0.07 | 7.29±0.06 | 7.30±0.06 | 7.25±0.10 | 0.68 |
| NICU admission | 19 (19%) | 3 (5.0%) | 11 (24%) | 0 | 0.03^*^ |

Supplementary Table 3. Multivariable linear regression: nausea severity in early pregnancy and psychological status

Multivariable linear regression models were used to examine the associations between nausea severity in early pregnancy and maternal psychological status. STAI-State score and PHQ-9 score in early pregnancy were used as dependent variables. Nausea severity was entered as an ordinal continuous variable based on subjective assessment (A = not troubled by nausea symptoms [0], B = symptoms without interference with daily activities [1], C = symptoms interfering with daily activities [2]). Covariates included maternal age, pre-pregnancy BMI, nulliparity, and history of psychiatric disorders. Data are presented as unstandardized regression coefficients with 95% confidence intervals, followed by p values.

BMI, body mass index; PHQ-9, Patient Health Questionnaire-9; STAI, State-Trait Anxiety Inventory

| Variable | STAI-State (early pregnancy) n = 224 | PHQ-9 (early pregnancy) n = 126 |
| --- | --- | --- |
| Nausea severity (A/B/C) | 4.295 [2.355, 6.235]  p = <0.001 | 3.622 [2.383, 4.862]  p = <0.001 |
| Age (years) | 0.111 [-0.136, 0.359]  p = 0.376 | -0.104 [-0.253, 0.045]  p = 0.171 |
| BMI (kg/m²) | 0.012 [-0.284, 0.308]  p = 0.935 | 0.061 [-0.128, 0.251]  p = 0.522 |
| Nulliparous | 3.713 [1.263, 6.164]  p = 0.003 | 0.847 [-0.709, 2.404]  p = 0.283 |
| Psychiatric history | 6.294 [2.807, 9.782]  p = <0.001 | 2.270 [-0.030, 4.571]  p = 0.053 |

**Supplementary Table 4. Maternal characteristics and perinatal outcomes according to the presence of nausea in early pregnancy based on subjective assessment**

Maternal characteristics and perinatal outcomes according to the presence or absence of nausea in early pregnancy based on subjective assessment. Among participants who underwent subjective evaluation of nausea in early pregnancy (n = 224), nausea status was categorized into two groups: “no symptoms” (category A) and “symptoms present” (categories B + C). Data are presented as mean ± standard deviation or n (%). (*p<0.05)

ART, assisted reproductive technology; BMI, body mass index; FGR, fetal growth restriction; GDM, gestational diabetes mellitus; HDP, hypertensive disorders of pregnancy; NICU, neonatal intensive care unit; PPH, postpartum hemorrhage

|  | No symptoms (n=30) | Symptoms present (n=194) | P value |
| --- | --- | --- | --- |
| Age | 33.6±4.5 | 34.3±5.0 | 0.46 |
| Pre-pregnancy BMI (kg/m^2^) | 21.3±3.0 | 22.3±4.4 | 0.48 |
| Gestational weight gain (kg) | 6.7±4.1 | 10.6±4.3 | 0.11 |
| primipara | 17 (57%) | 100 (52%) | 0.60 |
| ART | 9 (30%) | 63 (32%) | 0.79 |
| Psychiatric disorders | 4 (13%) | 30 (15%) | 0.76 |
| Gestational age at delivery (week) | 38.3±1.1 | 38.4±1.4 | 0.43 |
| Preterm birth | 0 | 9 (4.8%) | 0.22 |
| HDP | 7 (23%) | 25 (13%) | 0.15 |
| GDM | 6 (20%) | 13 (6.9%) | 0.02^*^ |
| FGR | 2 (6.7%) | 11 (5.9%) | 0.86 |
| Cesarean section | 12 (40%) | 69 (37%) | 0.73 |
| Emergency cesarean section | 2 (6.7%) | 9 (4.8%) | 0.67 |
| Intrapartum blood loss (mL) | 644±402 | 731±436 | 0.14 |
| PPH | 9 (30%) | 83 (44%) | 0.15 |
| Placental weight (g) | 511±88 | 561±98 | 0.35 |
| Birth weight (g) | 2988±401 | 2977±372 | 0.97 |
| Apgar score at 1 minute | 8.2±0.8 | 8.1±1.8 | 0.84 |
| Apgar score at 5 minutes | 9.3±0.5 | 9.3±0.6 | 0.81 |
| Umbilical artery blood pH | 7.30±0.07 | 7.29±0.06 | 0.39 |
| NICU admission | 4 (13%) | 30 (16%) | 0.72 |

**Supplementary Table 5. Maternal characteristics and perinatal outcomes according to the presence of nausea in early pregnancy based on the Emesis Index**

Maternal characteristics and perinatal outcomes in early pregnancy based on the Emesis Index. Among participants who underwent quantitative evaluation of nausea using the Emesis Index in early pregnancy (n = 212), nausea status was categorized into two groups: “no symptoms” (EI ≤3) and “symptoms present” (EI ≥4). Data are presented as mean ± standard deviation or n (%).

ART, assisted reproductive technology; BMI, body mass index; EI, emesis index; FGR, fetal growth restriction; GDM, gestational diabetes mellitus; HDP, hypertensive disorders of pregnancy; NICU, neonatal intensive care unit; PPH, postpartum hemorrhage

|  | No symptoms (n=101) | Symptoms present (n=111) | P value |
| --- | --- | --- | --- |
| Age | 35.0±4.9 | 33.6±5.0 | 0.06 |
| Pre-pregnancy BMI (kg/m^2^) | 21.7±3.5 | 22.8±4.9 | 0.29 |
| Gestational weight gain (kg) | 11.1±3.8 | 10.2±4.5 | 0.27 |
| primipara | 56 (55%) | 53 (48%) | 0.26 |
| ART | 35 (35%) | 37 (33%) | 0.84 |
| Psychiatric disorders | 16 (16%) | 16 (14%) | 0.77 |
| Gestational age at delivery (week) | 38.4±1.4 | 38.4±1.3 | 0.98 |
| Preterm birth | 5 (5.1%) | 4 (3.7%) | 0.62 |
| HDP | 14 (14%) | 17 (16%) | 0.77 |
| GDM | 8 (8.2%) | 10 (9.3%) | 0.78 |
| FGR | 5 (5.1%) | 7 (6.5%) | 0.67 |
| Cesarean section | 40 (41%) | 35 (32%) | 0.21 |
| Emergency cesarean section | 5 (5.1%) | 4 (3.7%) | 0.63 |
| Intrapartum blood loss (mL) | 670±362 | 742±473 | 0.46 |
| PPH | 36 (37%) | 51 (47%) | 0.49 |
| Placental weight (g) | 541±87 | 569±102 | 0.28 |
| Birth weight (g) | 2940±379 | 3007±371 | 0.16 |
| Apgar score at 1 minute | 8.1±1.2 | 8.2±1.1 | 0.55 |
| Apgar score at 5 minutes | 9.3±0.6 | 9.3±0.6 | 0.45 |
| Umbilical artery blood pH | 7.29±0.07 | 7.29±0.06 | 0.53 |
| NICU admission | 19 (19%) | 14 (13%) | 0.22 |

**Supplementary Table 6. Maternal characteristics and perinatal outcomes according to the presence of nausea in mid pregnancy based on subjective assessment**

Maternal characteristics and perinatal outcomes in mid pregnancy based on subjective assessment. Among participants who underwent subjective evaluation of nausea in mid pregnancy (n = 402), nausea status was categorized into two groups: “no symptoms” (category A) and “symptoms present” (categories B + C). Data are presented as mean ± standard deviation or n (%). (*p<0.05)

ART, assisted reproductive technology; BMI, body mass index; FGR, fetal growth restriction; GDM, gestational diabetes mellitus; HDP, hypertensive disorders of pregnancy; NICU, neonatal intensive care unit; PPH, postpartum hemorrhage

|  | No symptoms (n=307) | Symptoms present (n=95) | P value |
| --- | --- | --- | --- |
| Age | 34.3±5.2 | 32.8±5.1 | 0.03^*^ |
| Pre-pregnancy BMI (kg/m^2^) | 21.9±3.8 | 22.7±4.6 | 0.23 |
| Gestational weight gain (kg) | 10.6±3.9 | 10.2±5.1 | 0.47 |
| primipara | 165 (54%) | 48 (51%) | 0.58 |
| ART | 103 (34%) | 30 (32%) | 0.72 |
| Psychiatric disorders | 29 (9.5%) | 19 (20%) | 0.01^*^ |
| Gestational age at delivery (week) | 38.4±1.4 | 38.5±1.3 | 0.56 |
| Preterm birth | 13 (4.3%) | 4 (4.3%) | 0.98 |
| HDP | 40 (13%) | 13 (14%) | 0.88 |
| GDM | 30 (9.9%) | 6 (6.4%) | 0.30 |
| FGR | 19 (6.3%) | 7 (7.5%) | 0.68 |
| Cesarean section | 113 (37%) | 35 (37%) | 0.97 |
| Emergency cesarean section | 26 (8.7%) | 7 (7.5%) | 0.73 |
| Intrapartum blood loss (mL) | 761±613 | 795±584 | 0.52 |
| PPH | 136 (45%) | 47 (50%) | 0.40 |
| Placental weight (g) | 562±93 | 555±110 | 0.66 |
| Birth weight (g) | 2979±412 | 3000±404 | 0.75 |
| Apgar score at 1 minute | 8.1±1.0 | 8.0±1.2 | 0.47 |
| Apgar score at 5 minutes | 9.3±0.7 | 9.2±0.6 | 0.12 |
| Umbilical artery blood pH | 7.30±0.06 | 7.29±0.06 | 0.36 |
| NICU admission | 49 (16%) | 17 (18%) | 0.68 |

**Supplementary Table 7. Maternal characteristics and perinatal outcomes according to the presence of nausea in mid pregnancy based on the Emesis Index**

Maternal characteristics and perinatal outcomes in mid pregnancy based on the Emesis Index. Among participants who underwent quantitative evaluation of nausea using the Emesis Index in mid pregnancy (n = 360), nausea status was categorized into two groups: “no symptoms” (EI ≤3) and “symptoms present” (EI ≥4). Data are presented as mean ± standard deviation or n (%). (*p<0.05)

ART, assisted reproductive technology; BMI, body mass index; EI, emesis index; FGR, fetal growth restriction; GDM, gestational diabetes mellitus; HDP, hypertensive disorders of pregnancy; NICU, neonatal intensive care unit; PPH, postpartum hemorrhage

|  | No symptoms (n=330) | Symptoms present (n=30) | P value |
| --- | --- | --- | --- |
| Age | 34.2±5.1 | 33.4±4.7 | 0.62 |
| Pre-pregnancy BMI (kg/m^2^) | 22.1±4.2 | 22.4±3.8 | 0.52 |
| Gestational weight gain (kg) | 10.5±4.1 | 10.0±6.1 | 0.72 |
| primipara | 175 (53%) | 14 (50%) | 0.78 |
| ART | 108 (33%) | 6 (21%) | 0.23 |
| Psychiatric disorders | 37 (11%) | 6 (21%) | 0.11 |
| Gestational age at delivery (week) | 38.3±1.4 | 38.7±1.1 | 0.26 |
| Preterm birth | 16 (4.9%) | 0 | 0.23 |
| HDP | 44 (14%) | 2 (7.1%) | 0.34 |
| GDM | 30 (9.2%) | 1 (3.6%) | 0.31 |
| FGR | 24 (7.4%) | 1 (3.6%) | 0.45 |
| Cesarean section | 124 (38%) | 7 (25%) | 0.17 |
| Emergency cesarean section | 22 (6.8%) | 3 (11%) | 0.44 |
| Intrapartum blood loss (mL) | 762±627 | 771±396 | 0.18 |
| PPH | 139 (43%) | 18 (64%) | 0.03^*^ |
| Placental weight (g) | 561±97 | 555±111 | 0.73 |
| Birth weight (g) | 2976±415 | 3011±425 | 0.78 |
| Apgar score at 1 minute | 8.1±1.1 | 8.1±0.6 | 0.47 |
| Apgar score at 5 minutes | 9.3±0.7 | 9.2±0.5 | 0.15 |
| Umbilical artery blood pH | 7.30±0.06 | 7.30±0.06 | 0.75 |
| NICU admission | 56 (17%) | 6 (20%) | 0.55 |

**Supplementary Table 8. Maternal characteristics and perinatal outcomes according to the presence of nausea in late pregnancy based on subjective assessment**

Maternal characteristics and perinatal outcomes in late pregnancy based on subjective assessment. Among participants who underwent subjective evaluation of nausea in late pregnancy (n = 424), nausea status was categorized into two groups: “no symptoms” (category A) and “symptoms present” (categories B + C). Data are presented as mean ± standard deviation or n (%). (*p<0.05)

ART, assisted reproductive technology; BMI, body mass index; FGR, fetal growth restriction; GDM, gestational diabetes mellitus; HDP, hypertensive disorders of pregnancy; NICU, neonatal intensive care unit; PPH, postpartum hemorrhage

|  | No symptoms (n=324) | Symptoms present (n=100) | P value |
| --- | --- | --- | --- |
| Age | 34.0±5.0 | 33.8±5.1 | 0.84 |
| Pre-pregnancy BMI (kg/m^2^) | 21.8±4.1 | 22.1±4.2 | 0.59 |
| Gestational weight gain (kg) | 10.6±4.0 | 10.3±4.9 | 0.44 |
| primipara | 166 (51%) | 49 (49%) | 0.78 |
| ART | 9 (29%) | 24 (24%) | 0.36 |
| Psychiatric disorders | 27 (8.3%) | 15 (15%) | 0.04^*^ |
| Gestational age at delivery (week) | 38.6±1.2 | 38.6±1.0 | 0.82 |
| Preterm birth | 9 (2.8%) | 1 (1.0%) | 0.31 |
| HDP | 32 (10%) | 15 (15%) | 0.15 |
| GDM | 39 (12%) | 6 (6.0%) | 0.09 |
| FGR | 14 (4.4%) | 1 (1.0%) | 0.12 |
| Cesarean section | 113 (35%) | 38 (38%) | 0.53 |
| Emergency cesarean section | 27 (8.5%) | 13 (13%) | 0.16 |
| Intrapartum blood loss (mL) | 734±465 | 745±418 | 0.63 |
| PPH | 156 (49%) | 47 (47%) | 0.89 |
| Placental weight (g) | 560±99 | 560±96 | 0.95 |
| Birth weight (g) | 3012±388 | 3035±332 | 0.71 |
| Apgar score at 1 minute | 8.1±1.0 | 8.3±0.6 | 0.90 |
| Apgar score at 5 minutes | 9.4±0.6 | 9.4±0.5 | 0.74 |
| Umbilical artery blood pH | 7.30±0.06 | 7.30±0.06 | 0.53 |
| NICU admission | 41 (13%) | 12 (12%) | 0.88 |

**Supplementary Table 9. Maternal characteristics and perinatal outcomes according to the presence of nausea in late pregnancy based on the Emesis Index**

Maternal characteristics and perinatal outcomes in late pregnancy based on the Emesis Index. Among participants who underwent quantitative evaluation of nausea using the Emesis Index in late pregnancy (n = 386), nausea status was categorized into two groups: “no symptoms” (EI ≤3) and “symptoms present” (EI ≥4). Data are presented as mean ± standard deviation or n (%). (*p<0.05)

ART, assisted reproductive technology; BMI, body mass index; EI, emesis index; FGR, fetal growth restriction; GDM, gestational diabetes mellitus; HDP, hypertensive disorders of pregnancy; NICU, neonatal intensive care unit; PPH, postpartum hemorrhage

|  | No symptoms (n=362) | Symptoms present (n=24) | P value |
| --- | --- | --- | --- |
| Age | 34.1±4.9 | 33.0±5.6 | 0.57 |
| Pre-pregnancy BMI (kg/m^2^) | 21.9±4.1 | 21.6±2.9 | 0.65 |
| Gestational weight gain (kg) | 10.3±3.9 | 12.0±6.9 | 0.54 |
| primipara | 188 (52%) | 13 (54%) | 0.83 |
| ART | 111 (31%) | 3 (13%) | 0.06 |
| Psychiatric disorders | 35 (9.7%) | 4 (17%) | 0.27 |
| Gestational age at delivery (week) | 38.6±1.2 | 38.5±0.9 | 0.41 |
| Preterm birth | 9 (2.5%) | 0 | 0.43 |
| HDP | 39 (11%) | 3 (13%) | 0.82 |
| GDM | 38 (11%) | 3 (13%) | 0.78 |
| FGR | 14 (3.9%) | 0 | 0.32 |
| Cesarean section | 127 (36%) | 10 (42%) | 0.55 |
| Emergency cesarean section | 30 (8.5%) | 4 (17%) | 0.17 |
| Intrapartum blood loss (mL) | 735±466 | 740±338 | 0.40 |
| PPH | 170 (48%) | 13 (54%) | 0.54 |
| Placental weight (g) | 555±97 | 610±98 | 0.14 |
| Birth weight (g) | 3004±372 | 3168±387 | 0.05 |
| Apgar score at 1 minute | 8.2±1.0 | 8.0±0.6 | 0.08 |
| Apgar score at 5 minutes | 9.4±0.6 | 9.1±0.4 | 0.06 |
| Umbilical artery blood pH | 7.30±0.06 | 7.30±0.06 | 0.99 |
| NICU admission | 44 (12%) | 4 (17%) | 0.54 |

Supplementary Table 10. Multivariable linear regression: nausea presence at each gestational stage and psychological status (subjective assessment)

Multivariable linear regression models were used to examine the associations between nausea presence at each gestational stage and maternal psychological status. STAI-State and PHQ-9 scores at the corresponding gestational stage were used as dependent variables. Nausea presence was defined as subjective categories B or C, with category A as the reference. Covariates included maternal age, pre-pregnancy BMI, nulliparity, and history of psychiatric disorders. Data are presented as unstandardized regression coefficients with 95% confidence intervals, followed by p values.

BMI, body mass index; PHQ-9, Patient Health Questionnaire-9; STAI, State-Trait Anxiety Inventory

| Variable | Early pregnancy | | Mid-pregnancy | | Late pregnancy | |
| --- | --- | --- | --- | --- | --- | --- |
|  | STAI-State n = 224 | PHQ-9 n = 126 | STAI-State n = 399 | PHQ-9 n = 235 | STAI-State n = 422 | PHQ-9 n = 267 |
| Nausea present† | 5.557 [1.912, 9.202]  p = 0.003 | 4.200 [1.808, 6.593]  p = <0.001 | 4.187 [2.101, 6.273]  p = <0.001 | 1.879 [0.713, 3.046]  p = 0.002 | 1.922 [-0.082, 3.926]  p = 0.060 | 1.276 [0.282, 2.271]  p = 0.012 |
| Age (years) | 0.064 [-0.188, 0.317]  p = 0.618 | -0.068 [-0.284, 0.148]  p = 0.534 | 0.234 [0.058, 0.410]  p = 0.009 | 0.013 [-0.087, 0.114]  p = 0.793 | 0.141 [-0.032, 0.314]  p = 0.109 | 0.041 [-0.054, 0.136]  p = 0.395 |
| BMI (kg/m²) | 0.083 [-0.216, 0.382]  p = 0.585 | 0.103 [-0.121, 0.327]  p = 0.368 | 0.020 [-0.201, 0.241]  p = 0.860 | 0.015 [-0.099, 0.128]  p = 0.799 | 0.141 [-0.064, 0.347]  p = 0.178 | 0.010 [-0.090, 0.111]  p = 0.840 |
| Nulliparous | 3.717 [1.213, 6.221]  p = 0.004 | 0.654 [-0.806, 2.113]  p = 0.378 | 1.552 [-0.204, 3.307]  p = 0.083 | 0.393 [-0.599, 1.386]  p = 0.436 | 0.105 [-1.601, 1.810]  p = 0.904 | 0.455 [-0.397, 1.306]  p = 0.295 |
| Psychiatric history | 5.911 [2.351, 9.472]  p = 0.001 | 4.791 [2.598, 6.985]  p = <0.001 | 5.489 [2.676, 8.303]  p = <0.001 | 2.278 [0.815, 3.741]  p = 0.002 | 5.442 [2.546, 8.337]  p = <0.001 | 2.419 [1.199, 3.639]  p = <0.001 |

**Supplementary Table 11. Maternal characteristics and perinatal outcomes** according to the duration score of nausea based on subjective assessment

This analysis included participants who underwent continuous assessment of nausea throughout pregnancy (n = 134). The duration score (range, 0–3) was calculated by assigning one point for the presence of nausea at each gestational stage (early, mid, and late pregnancy), defined as subjective assessment categories B or C. Anxiety and depressive symptoms were assessed using the State–Trait Anxiety Inventory (STAI) and the Patient Health Questionnaire-9 (PHQ-9). Data are presented as mean ± standard deviation or n (%).

ART, assisted reproductive technology; BMI, body mass index; FGR, fetal growth restriction; GDM, gestational diabetes mellitus; HDP, hypertensive disorders of pregnancy; NICU, neonatal intensive care unit; PPH, postpartum hemorrhage

|  | 0point (n=14) | 1point (n=74) | 2point (n=28) | 3point (n=18) | P value |
| --- | --- | --- | --- | --- | --- |
| Age | 32.7±4.4 | 34.7±4.6 | 33.3±5.3 | 32.3±5.4 | 0.27 |
| Pre-pregnancy BMI (kg/m^2^) | 20.6±2.3 | 21.9±3.9 | 23.0±5.3 | 22.0±4.4 | 0.53 |
| Gestational weight gain (kg) | 10.6±3.5 | 10.5±4.1 | 10.5±3.6 | 10.4±6.0 | 0.78 |
| primipara | 7 (50%) | 43 (58%) | 16 (57%) | 11 (61%) | 0.93 |
| ART | 5 (36%) | 32 (43%) | 7 (25%) | 3 (17%) | 0.11 |
| Gestational age at delivery (week) | 38.5±1.2 | 38.5±1.2 | 38.8±1.2 | 39.1±1.1 | 0.20 |
| Preterm birth | 0 | 2 (2.9%) | 1 (3.6%) | 0 | 0.79 |
| HDP | 2 (14%) | 9 (13%) | 5 (18%) | 3 (17%) | 0.93 |
| GDM | 2 (14%) | 5 (7.1%) | 1 (3.6%) | 1 (5.6%) | 0.63 |
| FGR | 2 (14%) | 3 (4.3%) | 1 (3.6%) | 0 | 0.27 |
| Cesarean section | 5 (36%) | 23 (33%) | 7 (25%) | 5 (28%) | 0.85 |
| Emergency cesarean section | 0 | 5 (7.3%) | 2 (7.1%) | 0 | 0.49 |
| Intrapartum blood loss (mL) | 641±442 | 760±489 | 599±365 | 717±364 | 0.23 |
| PPH | 4 (29%) | 36 (51%) | 11 (39%) | 10 (56%) | 0.30 |
| Placental weight (g) | 552±90 | 565±99 | 539±80 | 576±118 | 0.43 |
| Birth weight (g) | 2923±386 | 2999±328 | 2992±269 | 3150±428 | 0.36 |
| Apgar score at 1 minute | 8.3±0.8 | 8.1±1.1 | 8.0±1.1 | 8.3±0.5 | 0.82 |
| Apgar score at 5 minutes | 9.4±0.6 | 9.3±0.6 | 9.3±0.5 | 9.2±0.4 | 0.83 |
| Umbilical artery blood pH | 7.29±0.09 | 7.29±0.06 | 7.29±0.08 | 7.30±0.06 | 0.63 |
| NICU admission | 1 (7.1%) | 10 (14%) | 3 (11%) | 2 (11%) | 0.89 |

**Supplementary Table 12. Maternal characteristics and perinatal outcomes** according to the duration score of nausea based on the Emesis Index

This analysis included participants who underwent continuous quantitative assessment using the Emesis Index (EI) throughout pregnancy (n = 134). The duration score (range, 0–3) was calculated by assigning one point for the presence of nausea at each gestational stage, defined as an EI score ≥4. Anxiety and depressive symptoms were evaluated using STAI and PHQ-9. Data are presented as mean ± standard deviation or n (%).

ART, assisted reproductive technology; BMI, body mass index; EI, emesis index; FGR, fetal growth restriction; GDM, gestational diabetes mellitus; HDP, hypertensive disorders of pregnancy; NICU, neonatal intensive care unit; PPH, postpartum hemorrhage

|  | 0point (n=58) | 1point (n=62) | 2point (n=8) | 3point (n=6) | P value |
| --- | --- | --- | --- | --- | --- |
| Age | 34.3±4.9 | 34.3±4.4 | 28.8±5.9 | 31.7±5.8 | 0.08 |
| Pre-pregnancy BMI (kg/m^2^) | 21.4±3.5 | 22.5±4.7 | 24.2±5.8 | 22.3±2.6 | 0.41 |
| Gestational weight gain (kg) | 11.2±3.8 | 10.1±3.7 | 9.0±8.1 | 12.4±5.5 | 0.36 |
| primipara | 35 (60%) | 33 (53%) | 5 (63%) | 4 (67%) | 0.81 |
| ART | 22 (38%) | 23 (37%) | 2 (25%) | 0 | 0.27 |
| Gestational age at delivery (week) | 38.8±1.1 | 38.4±1.4 | 38.9±0.8 | 38.8±1.2 | 0.46 |
| Preterm birth | 0 | 3 (5.0%) | 0 | 0 | 0.19 |
| HDP | 7 (13%) | 10 (17%) | 2 (25%) | 0 | 0.55 |
| GDM | 3 (5.4%) | 5 (8.3%) | 1 (13%) | 0 | 0.66 |
| FGR | 3 (5.4%) | 3 (5.0%) | 0 | 0 | 0.70 |
| Cesarean section | 15 (27%) | 24 (40%) | 0 | 1 (17%) | 0.07 |
| Emergency cesarean section | 3 (5.4%) | 4 (6.8%) | 0 | 0 | 0.80 |
| Intrapartum blood loss (mL) | 642±391 | 777±511 | 630±266 | 704±323 | 0.38 |
| PPH | 25 (45%) | 26 (43%) | 5 (63%) | 5 (83%) | 0.22 |
| Placental weight (g) | 543±88 | 567±101 | 540±112 | 617±99 | 0.47 |
| Birth weight (g) | 2963±301 | 3004±335 | 3151±288 | 3313±607 | 0.13 |
| Apgar score at 1 minute | 8.1±1.1 | 8.1±1.0 | 8.3±0.5 | 8.2±0.4 | 0.93 |
| Apgar score at 5 minutes | 9.3±0.5 | 9.3±0.6 | 9.4±0.5 | 9.2±0.4 | 0.89 |
| Umbilical artery blood pH | 7.29±0.08 | 7.29±0.06 | 7.29±0.08 | 7.31±0.04 | 0.84 |
| NICU admission | 7 (12%) | 6 (10%) | 2 (25%) | 1 (17%) | 0.66 |

Supplementary Table 13. Multivariable linear regression: nausea duration score and psychological status at each gestational stage (n = 134, subjective assessment)

Multivariable linear regression models were used to examine the associations between nausea duration score and maternal psychological status at each gestational stage. STAI-State and PHQ-9 scores at the corresponding gestational stage were used as dependent variables. The nausea duration score (0–3) was calculated by assigning one point for the presence of nausea at each gestational stage, defined as subjective categories B or C. Covariates included maternal age, pre-pregnancy BMI, nulliparity, and history of psychiatric disorders. Data are presented as unstandardized regression coefficients with 95% confidence intervals, followed by p values.

BMI, body mass index; PHQ-9, Patient Health Questionnaire-9; STAI, State-Trait Anxiety Inventory

| Variable | Early pregnancy | | Mid-pregnancy | | Late pregnancy | |
| --- | --- | --- | --- | --- | --- | --- |
|  | STAI-State n = 130 | PHQ-9 n = 80 | STAI-State n = 130 | PHQ-9 n = 126 | STAI-State n = 130 | PHQ-9 n = 130 |
| Nausea duration score (0–3)‡ | 2.403 [0.447, 4.359]  p = 0.016 | 2.018 [0.711, 3.325]  p = 0.003 | 3.275 [1.377, 5.174]  p = <0.001 | 1.603 [0.853, 2.353]  p = <0.001 | 1.477 [-0.518, 3.471]  p = 0.145 | 0.800 [0.097, 1.502]  p = 0.026 |
| Age (years) | 0.258 [-0.368, 0.884]  p = 0.417 | 0.030 [-0.324, 0.385]  p = 0.865 | 0.326 [-0.009, 0.662]  p = 0.057 | 0.014 [-0.118, 0.147]  p = 0.829 | 0.165 [-0.318, 0.648]  p = 0.500 | 0.068 [-0.074, 0.211]  p = 0.345 |
| BMI (kg/m²) | -0.178 [-0.836, 0.481]  p = 0.594 | 0.110 [-0.261, 0.481]  p = 0.557 | -0.135 [-0.524, 0.253]  p = 0.492 | 0.083 [-0.070, 0.236]  p = 0.285 | -0.090 [-0.597, 0.417]  p = 0.727 | -0.038 [-0.188, 0.111]  p = 0.614 |
| Nulliparous | 4.086 [-0.173, 8.345]  p = 0.060 | 1.264 [-0.983, 3.511]  p = 0.268 | 1.804 [-1.378, 4.985]  p = 0.264 | 0.592 [-0.675, 1.860]  p = 0.357 | 1.616 [-1.658, 4.891]  p = 0.331 | 0.622 [-0.342, 1.586]  p = 0.204 |
| Psychiatric history | 10.186 [4.965, 15.407]  p = <0.001 | 5.527 [2.378, 8.676]  p = 0.001 | 7.550 [2.847, 12.254]  p = 0.002 | 2.018 [0.168, 3.868]  p = 0.033 | 6.252 [2.490, 10.014]  p = 0.001 | 2.590 [1.477, 3.702]  p = <0.001 |

**Supplementary Table 14. Emesis Index (EI): components and scoring system**

The Emesis Index (EI) is a quantitative symptom score assessing nausea and vomiting during pregnancy. The EI consists of five items evaluating nausea, vomiting, appetite loss, salivation, and oral discomfort, each scored from 0 to 3 according to symptom frequency or severity. The total EI score is calculated as the sum of individual item scores, with higher scores indicating more severe symptoms.

| **Symptom** | **Score 0** | **Score 1** | **Score 2** | **Score 3** |
| --- | --- | --- | --- | --- |
| **Nausea** | None | 1–4 times/day | 5–10 times/day | Constant |
| **Vomiting** | None | 1–4 times/day | 5–10 times/day | Constant |
| **Loss of appetite** | None | Mild | Moderate (30–40% intake) | Severe (≤20% intake) |
| **Salivation** | None | Slightly increased | Increased but tolerable | Markedly increased and distressing |
| **Oral discomfort** | None | Slightly increased | Increased but tolerable | Markedly increased and distressing |
